# Does the Distance Between Ground Poles Affect Limb, Spinal and Pelvic Kinematics in Horses When Walking In-Hand?

**DOI:** 10.3390/ani16131938

**Published:** 2026-06-23

**Authors:** Lucy Douglas, Christy Maddock, Ronja Parker, Russell MacKechnie-Guire, Vicki Walker

**Affiliations:** Equine Department, Hartpury University, Hartpury, Gloucestershire GL19 3BE, UKronja.parker@hartpury.ac.uk (R.P.);

**Keywords:** equine biomechanics, polework, locomotion, rehabilitation, gait analysis, thoracolumbar spine, limb range of motion, training

## Abstract

Polework exercise is commonly used in equine training and rehabilitation; however, the influence of pole spacing on locomotor kinematics remains unclear. This study investigated the effects of pole spacing on limb, thoracolumbar and pelvic kinematics in horses walking in-hand. Eleven warmblood horses were assessed during overground walking and over five ground poles set at 85%, 100% and 105% of forelimb step length. Pole spacing did not significantly alter maximum fore- or hindlimb retraction; however, protraction–retraction range of motion (ROM) was reduced at 85% spacing. Forelimb ROM was reduced by 6.5% compared with 100% spacing and by 8.7% compared with 105% spacing, while hindlimb ROM was reduced by 2.9%. Hindlimb protraction was reduced at 85% compared with 100% and 105% spacing (*p* = 0.022, *p* = 0.034). Tarsal ROM increased over poles from NP: 38.0 ± 3.6°, poles: 62.1–64.2° (*p* < 0.001), irrespective of spacing. Walking over poles increased thoracic ROM at T10 from 7.9 ± 1.2° to 9.4–9.6° and at T13 from 8.7 ± 1.3° to 9.4–10.1° (*p* < 0.001), primarily through increased extension. Shorter spacing (85%) reduced T10 ROM compared with 100% spacing (*p* = 0.003). Changes in lumbar flexion were smaller, with significant effects observed only at L3 and L5, where maximal flexion increased from 9.6 ± 1.4° to 10.3 ± 1.8° and from 9.2 ± 1.5° to 9.9 ± 1.7°, respectively, at longer pole spacings (*p* < 0.05). These findings suggest that modest changes in pole spacing result in relatively small alterations in limb and thoracolumbar kinematics during walking. This should be considered when using polework in practice.

## Simple Summary

Ground poles are widely used in horse training and rehabilitation, but there is limited scientific evidence on how the distance between poles affects the way horses move. This study investigated how different pole spacings influence limb and back movement in horses walking in-hand. Horses walked overground and over five poles set at shorter, normal, and slightly longer distances based on their natural step length. The results showed that shorter pole spacing slightly reduced how far the limbs moved forward and backward, particularly in the hindlimbs. In contrast, all pole conditions increased movement at the hock (tarsal) joint. Walking over poles also affected the horse’s back, with longer pole distances increasing extension (hollowing) of the mid-back, while shorter distances reduced this effect. Changes in flexion (rounding) of the back were small. These findings show that pole spacing can influence both limb and back movement. As a result, the distance between poles should be considered when using polework as part of a training or rehabilitation programme.

## 1. Introduction

Hand-walking is the most commonly used rehabilitation modality, reported in 97% of rehabilitation cases [1], particularly during early-stage recovery when controlled loading is required to support tissue healing and reduce reinjury risk [2]. Walking exercise allows early rehabilitation priorities, such as increasing limb range of motion (ROM) and proprioception, to be addressed before progressing to strength and endurance development [3]. Different exercise methods are often prescribed as part of a multimodal programme to support the horse’s recovery [4], and whilst there is a growing body of evidence, there are still limited studies on how different exercise methods influence equine locomotion.

One method that is frequently utilised in equine training and rehabilitation is polework exercise, due to its proposed benefits for strength development, neuromotor control and proprioception [5,6]. Existing studies support the use of poles for increasing limb ROM at walk [7] and trot [8] without increasing vertical ground reaction force (GRF) [9] or midstance fetlock extension [7,8]. This is particularly relevant in rehabilitation contexts, where controlling limb loading may be desirable in healing tissues [2].

To successfully negotiate a ground pole, the horse must elevate its hoof to an appropriate height to ensure clearance [8], which is proposed to present both visuomotor coordination and proprioceptive feedback to facilitate coordinated limb placement dependent on the pole set up [6]. During this process, as a limb undergoes flexion, the contralateral limb(s) remains weight-bearing, which likely necessitates the activation of specific muscle groups to maintain balance and trunk support. Surface electromyography (sEMG) studies have demonstrated increases in peak activity of the longissimus dorsi and peak and average activity of the rectus abdominis during walking and trotting over ground poles [10]. Fine-wire EMG quantified increased multifidus activity at T12 and L5 during trotting over ground poles [11], both studies indicating an increase in core muscle activity. As the horse’s limbs flex and pass over the pole, it seems likely that this increase in core muscle activity may be a response of increased spinal mobility during polework, therefore acting to stabilise the axial segments. Studies have demonstrated greater ROM of the thoracolumbosacral spine in horses walking in-hand and during ridden exercise when trotting over poles [12,13]. Training and rehabilitation methods that promote a greater ROM of the thoracolumbosacral spine, moderated by an activation of the hypaxial and deep postural musculature, are frequently desirable. Although it remains unknown if the increased ROM is achieved via increased flexion or extension of the thoracolumbosacral spine. Studies which have measured increased thoracolumbar ROM over poles have utilised inertial measurement units (IMUs) to quantify spinal kinematics. IMUs are a validated and valuable method for field-based research [14,15]; however, a limitation is their inability to provide specific information on spinal position and therefore maximal flexion and extension during polework exercise. Characterising the mechanisms underlying changes in spinal mobility (i.e., whether increases are due to flexion, extension, or a combination of both) is important for exercise prescription, particularly in horses recovering from spinal pathology who are commonly prescribed polework exercise post-surgery [16].

Although polework is widely advocated in both training and rehabilitation contexts [5], there remains a paucity of quantitative data describing how inter-pole distance influences equine biomechanics. Variations in the spacing between poles are likely to have important biomechanical consequences, meaning that the success of task execution may depend significantly on how the poles are configured. Anecdotally, riders and trainers may use polework to encourage gait shortening and lengthening of the gait, but the distances used for pole spacing is usually set based on arbitrary guidelines [17] rather than evidence-based effects on locomotor patterns. Clayton [18] demonstrated that stride length varies systematically between collected, medium and extended walk, with collected and extended walk corresponding to approximately 85% and 105% of medium walk stride length using 2D videography, but this study did not quantify other limb or spinal kinematic variables.

Whilst limb kinematics during polework exercise have been reported, there is no information on how polework alters fore- and hindlimb protraction and retraction compared to overground walking and how this is influenced by pole spacing. The modified bow and string theory, presented by van Weeren et al. [19], suggests that movements which take the limbs away from the body in the sagittal plane (i.e., forelimb protraction and hindlimb retraction) may act to increase extension of the spine, whereas movements which put the limbs further under the trunk (i.e., forelimb retraction and hindlimb protraction) result in spinal flexion. In order to assess the effect of pole spacing on this interaction, we selected spacing distances not only because they correspond to collected and extended walk, but also because they represent moderate deviations from the horse’s preferred walking stride that are expected to require meaningful adjustments in limb protraction and retraction while remaining within the normal functional range of the walk. Shorter spacing (85%) may be expected to promote increased limb flexion and more regulated limb protraction, whereas longer spacing (105%) requires greater limb protraction and retraction to facilitate the increase in step length. Using these spacings therefore allowed investigation of locomotor adaptations to both shortened and lengthened stepping demands while minimising the likelihood of gait disruption or transition to another gait. The study aimed to compare the effects of ground pole (GP) spacing of 85%, 100% and 105% of step length on limb, spinal, and pelvic kinematics in horses walking in-hand, and it was hypothesised that:

1. Walking over ground poles will alter limb, spinal, and pelvic kinematics compared to overground locomotion.
2. Increasing pole spacing (105%) will result in greater limb, spinal, and pelvic range of motion compared to reduced spacing (85%).

## 2. Materials and Methods

### 2.1. Sample Size Calculation

Sample size calculations were performed using SPSS (Version 29) based on data reported by Walker et al. [7] for mid-swing tarsal flexion during walking over ground poles compared with overground locomotion. Using a significance level of 0.05 and a target power of 0.90, calculations indicated that a minimum of four horses would be required to detect a statistically significant difference. This relatively small sample size reflects the large effect size (>1) reported previously for tarsal flexion during polework [7]. However, the present study aimed to investigate a broader range of kinematic outcomes, including limb, spinal and pelvic variables, for which effect sizes were expected to be smaller and biological variability potentially greater, so 11 horses were used.

### 2.2. Horses

Eleven horses (mean ± SD, height: 168 ± 8 cm, age: 9 ± 3 years, eight mares and three geldings), mixed breed (warmblood and warmblood cross), were recruited. All were in regular training as allrounders (n = 3), competing in dressage (n = 2), show jumping (n = 4) or eventing (n = 2) (ridden at least 4 days a week), which included polework at least once a month. All had experience of polework both in-hand and ridden. Horses were deemed fit to take part, i.e., in regular work and deemed sound by their owner, and this was verified by a veterinary physiotherapist after a subjective gait evaluation.

### 2.3. Data Capture

**Figure 1:**
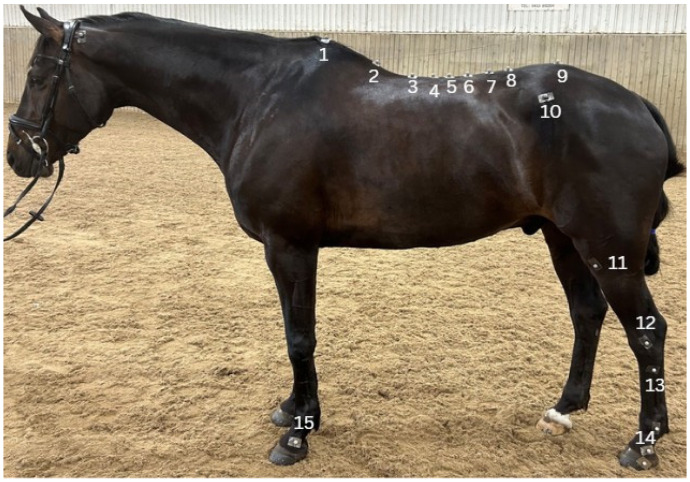
Fourteen-millimetre reflective markers were applied over the 6th (1), 10th (2), 13th (3), 15th (4) and 17th (5) thoracic and 1st (6), 3rd (7) and 5th (8) lumbar vertebrae and between the left and right tuber sacrale (9), as well as the left (10) (and right) tuber coxae, the lateral aspect of the tibial crest (11), the mid-talus (12), the third metatarsal bone at the junction with the base of the fourth metatarsal bone (13), and the lateral aspect of the coronary band over the collateral ligament for the fore- (15) and hindlimbs (14). Only markers used for analysis are defined here.

**Figure 2:**
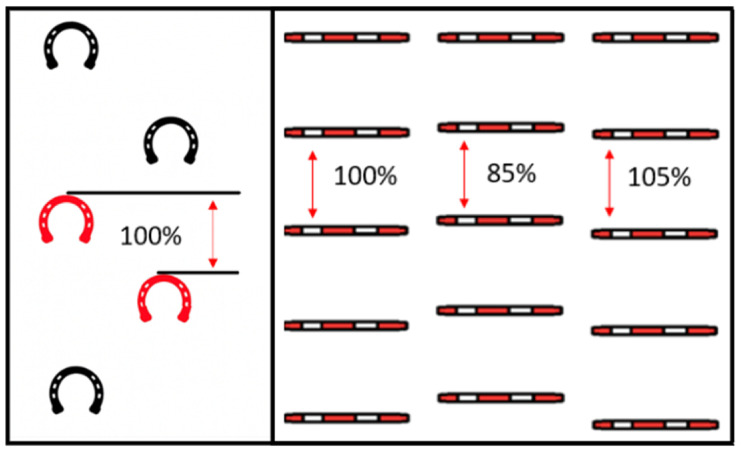
Pole set up for data collection with 5 ground poles (10 cm) set at 85%, 100% and 105% of baseline step length measured using a tape measure by the same researcher at the toe of consecutive forelimb hoof prints. Poles were measured at both ends and centre to centre when spacing was set.

Reflective markers (14 mm) were applied to the horse by the same experienced researcher (VW), using double-sided tape, on the dorsal midline at thoracic (T)6, T10, T13, T15, and T17; L1, L3, and L5; and between the left and right tuber sacrale (TS) and the left and right tuber coxae. Limb markers were positioned on the lateral collateral ligament of the distal interphalangeal joint of both forelimbs and hindlimbs. Additional hindlimb markers were applied to the proximal third metatarsal bone (at the junction with the fourth metatarsal base), mid-talus, and the lateral tibial crest and over the lateral collateral ligament at the coronary band (Figure 1). The horses were warmed up for ten minutes in-hand, leading from the left-hand, and the baseline step length (100%) was obtained by measuring the distance between left forelimb footfalls during three passes of medium walk (Figure 1) distances, with the measurements made by the same researcher using a tape measure. Ten optical motion-capture cameras (Miqus M3; Qualisys AB, Gothenburg, Sweden (240 Hz)) captured horses walking in-hand on a straight-line track marked out with cones (26.0 m× 1.8 m). The calibrated volume was 10 m × 2 m, with a standard deviation of wand length of 0.9 ± 0.0 mm and an average residual of 1.0 ± 0.2 mm. Four passes (two from the left, two from the right) were collected at four different conditions: no poles (NP) and five ground poles (10 cm) set at 85%, 100% and 105% of baseline step length in a randomized cross-over design (Figure 2). Pole distances were 80.5 cm ± 8 cm for 85%, 94.6 cm ± 9 cm for 100% and 99.5 cm ± 10 cm for 105%. Speed was controlled within 0.2 m/s within conditions. Any pass where the horse had more than one step between the poles, touched or knocked the pole was disregarded and the condition repeated. A maximum of 2 trials were repeated per horse per condition, and these occurred at 85% or 105%, with only one horse at each distance.

### 2.4. Data Analysis

Marker trajectories were digitised in Qualisys Track Manager (Qualisys AB, Gothenburg, Sweden; v2024) and subsequently exported for analysis. Kinematic marker X, Y, Z coordinates were imported into MATLAB (v2024, MathWorks Inc., Natick, MA, USA) for processing. All marker trajectories were filtered using a fourth-order Butterworth low-pass filter with a 10 Hz cut-off frequency. Strides were segmented using the velocity of the coronary band marker, with stride cycles defined between consecutive stance phases and processed separately for the fore- and hindlimbs.

**Figure 3:**
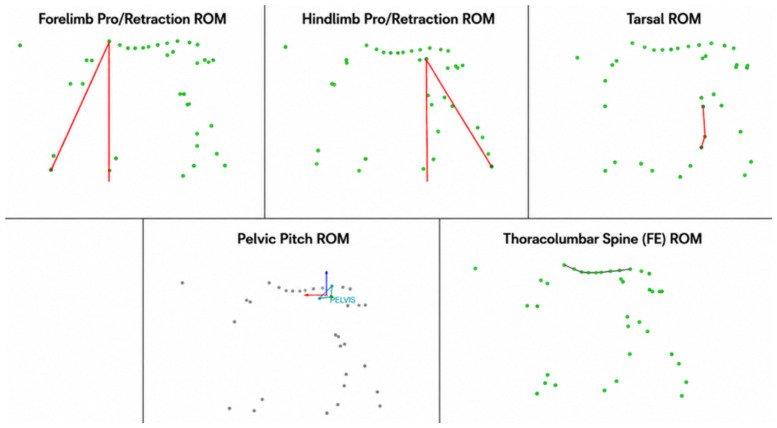
Images from Qualisys Track Manager (v2024) showing markers used to derive kinematic variables; forelimb protraction and retraction calculated from a vertical line from T6 and a line over the lateral collateral ligament at the coronary band, as well as hindlimb protraction and retraction from a vertical line from the tuber coxae (left or right) and over the lateral collateral ligament at the coronary band. Tarsal ROM was calculated using the markers placed on the proximal aspect of the third metatarsal bone at the junction with the base of the fourth metatarsal bone, the mid-talus, and the lateral aspect of the tibial crest. The pelvis was defined as a rigid body from the sacrum, left and right tuber coxae markers. Spinal kinematics analysis used methods published by Faber et al. [20], which use marker triplets that are named as per the centre marker, which is listed in bold (T6-**T10**-T13, T10-**T13**-T15, T15-**T18**-L1, T18-**L3**-L5, and L3-**L5**-TS), to calculate minimum and maximum amplitude and range of motion.

The spinal kinematics assessment used methods published by Faber et al. [20], which use marker triplets to calculate maximal extension (AMP Min), maximal flexion (AMP Max) and range of motion (ROM). The location is named as per the centre marker, which is listed in bold (T6-**T10**-T13, T10-**T13**-T15, T15-**T18**-L1, T18-**L3**-L5, and L3-**L5**-TS). Pelvic kinematics were defined using a rigid-body model based on markers placed over the left and right tuber coxae and the tubera sacrale (Figure 3).

Limb kinematics were calculated in the sagittal plane. Tarsal angle was calculated using markers 11, 12, and 13 (Figure 2). Hindlimb protraction and retraction angles were calculated as the angle between a vertical line passing through the tuber coxae and a line connecting the tuber coxae to the hindlimb coronary band marker. Forelimb protraction and retraction angles were calculated as the angle between a vertical line from T6 and a line connecting T6 to the forelimb coronary band marker (Figure 3).

For each stride, limb protraction, retraction and tarsal joint kinematics were computed, and minimum angle, maximum angle and the difference between the min and max were used to calculate range of motion (ROM), obtained on a per-stride basis. All per-stride limb, pelvic and spinal kinematic outcomes were exported to SPSS (v29) for statistical analysis.

### 2.5. Statistical Analysis

Descriptive statistics were carried out for each variable in each condition: NP and GPs set at 85%, 100% and 105% of baseline step length. A Shapiro–Wilk test indicated that the data were normally distributed therefore paired *t*-tests were used to compare the left and right sides of the horse for limb variables, but no significant differences were observed, so observations were pooled and a mean calculated. Linear mixed-effects models were used to investigate the relationship between outcome variables, limb, pelvic and spinal kinematics, with the predictor condition (pole distance) as a fixed effect and speed included as a covariate. No poles were set as the baseline condition in all models (limb, pelvic and spinal) (Supplementary Tables S1 and S2). Horse was used as a random effect term to control for the multiple (clustered) measures that were taken from each horse. Post hoc tests compared pole conditions. A Bonferroni correction for multiple comparisons was applied in SPSS, and adjusted *p*-values are reported. A significance value of *p* < 0.05 was applied in all analyses.

## 3. Results

### 3.1. Maximum Fore- and Hindlimb Protraction and Retraction and Range of Motion

When walking over poles at any distance, maximal fore- and hindlimb protraction and retraction, and ROM were not significantly altered compared with overground values (NP) (*p* > 0.05—see Supplementary Table S1).

**Figure 4:**
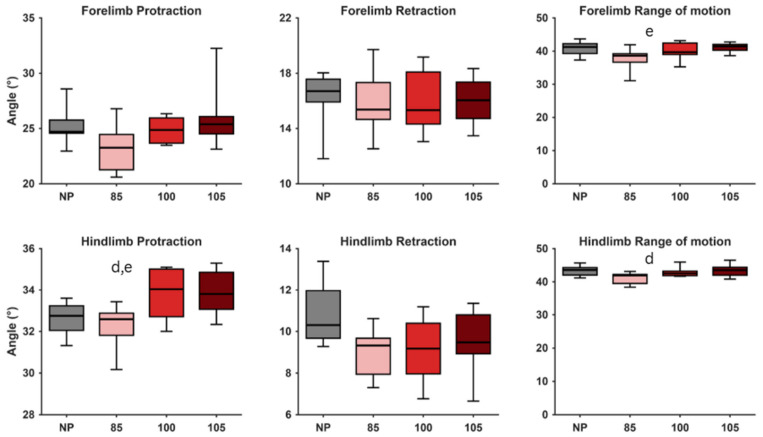
Boxplots displaying fore- and hindlimb maximum protraction, retraction and range of motion for each condition: no poles (NP) (red), 85% of step length (light grey), 100% of step length (medium grey), and 105% of step length (dark grey). The medians are represented by the black lines within the boxes. The boxes represent the 25th and 75th quartiles, and the whiskers indicate the most extreme data points, excluding outliers. Significant differences between conditions are indicated in superscript: d = between 85% and 100%, e = between 85% and 105%.

Forelimb protraction–retraction ROM was smaller at 85% pole spacing compared with 105% (*p* = 0.016). In the hindlimbs, shorter pole spacing (85%) resulted in reduced maximal hindlimb protraction compared with 100% and 105% spacing (*p* = 0.022 and *p* = 0.034, respectively) and a reduced hindlimb ROM compared with 100% (*p* = 0.015). Forelimb protraction and retraction and hindlimb protraction and retraction did not differ significantly between conditions (*p* > 0.05—see Supplementary Table S1) (Figure 4).

### 3.2. Tarsal Angle

**Figure 5:**
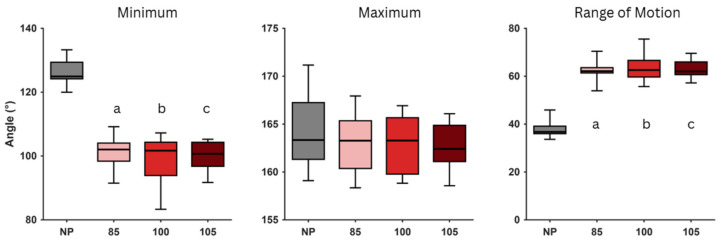
Boxplots displaying tarsal minimum, maximum and range of motion for each condition: no poles (NP) (red), 85% of step length (light grey), 100% of step length (medium grey), and 105% of step length (dark grey). The medians are represented by the black lines within the boxes. The boxes represent the 25th and 75th quartiles, and the whiskers indicate the most extreme data points, excluding outliers. Significant differences between conditions are indicated in superscript: a = between NP and 85%, b = between NP and 100%, c = between NP and 105%.

Tarsal swing flexion (min) and tarsal ROM were significantly greater over all pole conditions compared to NP (*p* < 0.001 for all). Maximum tarsal extension (max) was not significantly different between conditions (*p* > 0.05) (Figure 5). Tarsal min, max and ROM were not significantly different between pole spacings (*p* > 0.05) (Figure 5). See Supplementary Table S1 for individual *p*-values.

### 3.3. Spinal Kinematics

#### 3.3.1. Poles vs. No Poles

**Figure 6:**
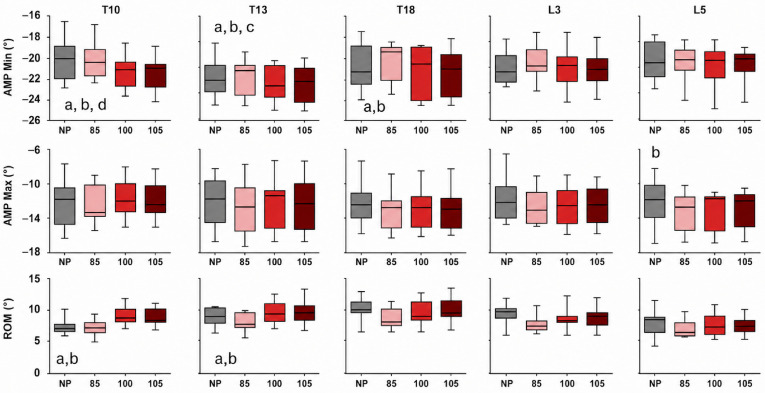
Boxplots displaying minimum, maximum and range of motion for T10, T13, T18, L3 and L5: no poles (NP) (red), 85% of step length (light grey), 100% of step length (medium grey), and 105% of step length (dark grey). AMPmax and AMPmin were calculated as the maximum and minimum values, respectively, of the stride-normalised kinematic waveform within a stride cycle. Range of motion (ROM) was calculated as the difference between AMPmax and AMPmin. Significant differences between conditions are indicated in superscript: a = between NP and 85%, b = between NP and 100%, c = between NP and 105%, d = between 85% and 100%. The medians are represented by the black lines within the boxes. The boxes represent the 25th and 75th quartiles, and the whiskers indicate the most extreme data points, excluding outliers.

The magnitude of these changes can be seen in Figure 6 and Table 1. Range of motion at T10 increased over all pole conditions compared to NP (*p* < 0.001).

At T13, ROM was increased over the 100% and 105% pole spacing compared to NP (*p* < 0.001).

No differences in ROM at T18, L3 or L5 were seen over poles compared to NP (*p* > 0.05) (Figure 6, Table 1).

Maximal extension angles at T10 and T18 increased over 100% and 105% pole spacing compared to NP (T10: *p* < 0.001, *p* = <0.001; T18: *p* = 0.015, *p* = 0.046). Maximal extension angle at T13 increased over all pole conditions compared to NP (*p* < 0.001). No significant differences were seen in maximum extension at L3 and L5 in any condition (*p* > 0.05).

It was observed that maximum flexion was decreased at L3 when walking over poles at 100% and 105% pole spacing compared to NP (*p* = 0.010 and *p* = 0.012, respectively), whereas max flexion at L5 was decreased at all pole distances compared to no poles (85%: *p* = 0.022, 100%: *p* = 0.006, 105%: *p* = 0.015). No significant effects on maximal flexion were seen at T10, T13 or T18 between conditions (*p* > 0.05).

#### 3.3.2. Pole Spacing

Poles set at 85% spacing reduced ROM at T10 via extension compared to 100% pole spacing (*p* = 0.003; *p* = 0.024). No significant effects on maximal flexion were seen at T10, T13, T18, L3 or L5 between pole spacing conditions (*p* > 0.05). The magnitude of these changes can be seen in Figure 6 and Table 1, and for individual *p*-values see Supplementary Table S1.

### 3.4. Pelvic Pitch ROM

**Figure 7:**
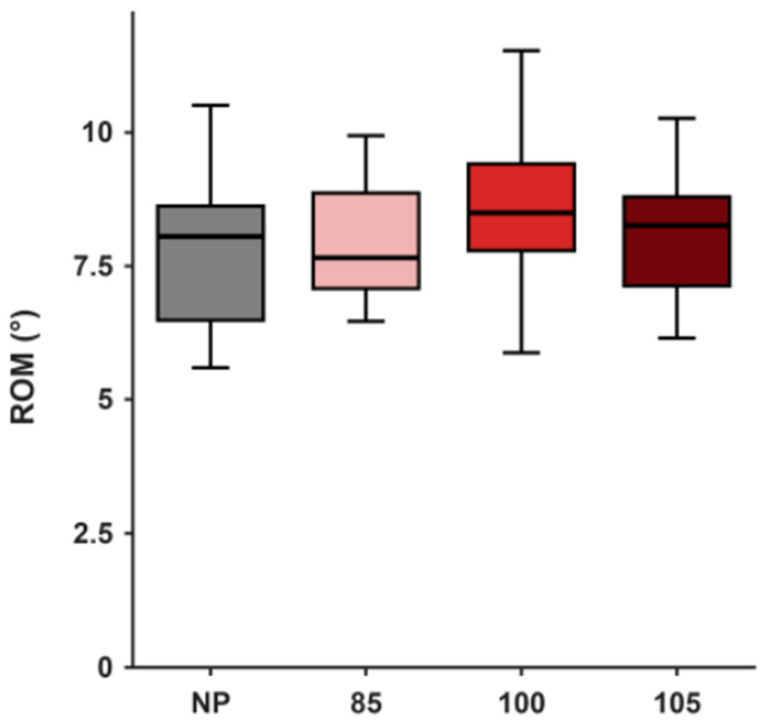
Pelvic pitch ROM: no poles (NP) (red), 85% of step length (light grey), 100% of step length (medium grey), and 105% of step length (dark grey). The medians are represented by the black lines within the boxes. The boxes represent the 25th and 75th quartiles, and the whiskers indicate the most extreme data points, excluding outliers.

No significant differences were seen in pelvic pitch ROM in any condition (*p* > 0.05) (Figure 7).

## 4. Discussion

This study aimed to quantify limb, spinal, and pelvic kinematics in horses walking in-hand overground and over five ground poles set at 85%, 100%, and 105% of forelimb step length. The first objective was to determine the effect of ground pole exercise compared to overground walking (NP), while the second was to evaluate how variations in pole spacing influenced these kinematic responses.

### 4.1. Poles vs. No Poles

The first hypothesis was partially supported, as walking over poles altered some, but not all, aspects of limb, spinal, and pelvic kinematics compared to overground walking (NP). Forelimb and hindlimb maximum protraction and retraction and total ROM were not significantly different between conditions, indicating that overall limb excursion in the sagittal plane was unaffected over poles. However, tarsal kinematics were altered, with increased flexion and ROM observed across all pole conditions compared to NP. In this study, tarsal ROM was used as a proxy for limb excursion, and our findings support those of previous work which identified increased tarsal ROM, via swing-phase flexion, in horses walking [7] and trotting [8] over poles.

In addition to limb effects, changes in spinal kinematics were also observed over poles compared to NP. Walking over poles increased mid-thoracic ROM, particularly at T10, where ROM was greater compared to overground walking regardless of pole spacing and at T13 and T18 for 100% and 105% spacing only. Reduced maximum flexion was observed at L3 and L5. Reduced flexion was seen over 100 and 105% at both locations and at 85% at L3 only. Whilst only a small reduction in maximal flexion was observed, these findings do not support anecdotal claims in the lay press that polework enhances spinal flexion. Nevertheless, the magnitude of the effect was small, and further work is needed to determine the clinical and functional relevance of this finding. Previous studies have identified a mobilising effect of ground and raised poles on the thoracolumbar spine [12,21,22], and the findings of this study supports this.

In the literature, the relationship between limb protraction and retraction and spinal flexion–extension was proposed by van Weeren et al. [19]. This built on the original bow and string theory by adding the role of the limbs. The current findings differ from the principles outlined by Van Weeren et al., but it is accepted that spinal kinematics at walk are passive and driven primarily by limb movements [23]. However, it is widely acknowledged that there are limitations to the bow and string theory (i.e., lack of head and neck), and the findings here may suggest that limb elevation also drives spinal kinematics at walk, but this is a hypothesis that requires further exploration in future studies.

Pelvic pitch ROM was unaltered when walking over poles compared to NP, and increased mediolateral displacement of the pelvis has been observed in previous work [7], potentially indicating that assessing pelvic translation and rotation in all planes is warranted in future work to fully understand the effects on pelvic motion.

### 4.2. Pole Spacing

Altering pole spacing influenced fore- and hindlimb protraction and ROM values but did not alter maximal fore- or hindlimb retraction. As hypothesised, a reduction in pole spacing at 85% of step length reduced forelimb ROM compared to 105% and hindlimb ROM compared to 100%. Maximal hindlimb protraction was smaller at 85% pole spacing compared with 100% and 105% spacing, but maximum forelimb protraction was unchanged between conditions.

These findings suggest that modest changes in pole spacing induce relatively small changes in fore- and hindlimb protraction–retraction ROM. At 85% spacing, forelimb ROM was reduced by 6.5% compared with 100% spacing and by 8.7% compared with 105% spacing, while hindlimb ROM was reduced by 2.9%. The reduction in hindlimb ROM was primarily driven by decreased maximal protraction. As maximal hindlimb protraction is often associated with hindlimb engagement, a characteristic frequently considered desirable in locomotor training and rehabilitation [24,25], the reduction observed at shorter pole spacings may be an important practical consideration when selecting pole distances. Pole spacing is frequently recommended anecdotally to encourage stride length adjustments [26]. The distances used in this study were based on stride length changes reported by Clayton [18], representing reductions in stride length consistent with collected walk (85%) and increases consistent with extended walk (105%). However, protraction–retraction variables were not reported, limiting direct comparison. The present findings therefore suggest that while spacing can influence limb kinematics, its effects are modest, and pole distances may be selected pragmatically according to specific training or rehabilitation goals.

Tarsal ROM was significantly greater over all pole conditions compared to NP, suggesting that walking over poles increases tarsal ROM via increased swing-phase flexion irrespective of pole spacing. In all cases, this was induced via an increase in maximal swing flexion, and our findings suggest that this effect is observed irrespective of modest changes in pole spacing. The use of different exercise methods for training and/or rehabilitation often utilises a multimodal approach, and currently polework, water treadmill, and the use of tactile stimulation or weighted boots can be considered to induce similar outcomes (i.e., increasing limb ROM). The findings reported here support the previous suggestions [7,27] that all are effective at increasing tarsal ROM. The increases in tarsal ROM appear to be greater whilst walking over 10 cm ground poles, in this study (~30°) and in Walker et al. [7] (~40°), compared to increases seen walking in 7.5 cm and 21 cm depth water (14° and 27° respectively) by Tranquille et al. [28], although in the water treadmill study, individual horses reached peak tarsal ROM at different water heights, suggesting that individual horse features need to be considered in terms of outcomes on limb ROM in practice. Tactile stimulators can also be used to increase hoof flight and limb flexion. Peak tarsal flexion increased relative to the no-stimulator condition by 20.5° with a tactile stimulator alone, 28.8° with limb weights, and 31.7° when limb weights were combined with tactile stimulation [29]. One consideration with these methods is that the horse habituates to their effects, with limb elevation decreasing after 10 consecutive strides [30]. It is important to acknowledge that these studies all used different methods, so future work comparing these options in the same population with consistent marker sets and methods would allow direct comparison. Within-study effects in previous work suggest that a greater pole height may have a greater influence than spacing on tarsal ROM [7]; however, larger spacing changes should be investigated in future work to assess their implications.

Pole spacing did influence spinal kinematics, with walking over poles increasing ROM at T10 and T13, but when the poles were set at the 100% and 105% step lengths, this increase in ROM was primarily driven by greater extension. Maximal extension at T18 was increased at these distances. Although this did not influence overall ROM at T18, these findings suggest that walking over ground poles at 100% and 105% spacing will increase extension of the mid- and caudal thoracic spine. Both ROM and maximal extension at T10 were reduced at 85% spacing compared to 100%, with these findings suggesting that shorter pole spacing may moderate thoracic extension. The optical motion-capture system used in the present study has previously been shown to have a positional accuracy of 0.58 mm, corresponding to approximately 0.5–3% of the measured range of motion [14]; additionally, skin-mounted markers have been validated against bone-fixed markers for the assessment of equine thoracolumbar kinematics [20]. Although between-session variability has also been quantified [31], these estimates are not directly applicable to the present study, as all measurements were collected within a single session. Sources of error associated with soft tissue artefacts and skin-marker placement remain and should be considered when interpreting small differences in spinal kinematic variables.

### 4.3. Practical Implications

An important consideration when selecting exercise for an individual horse is its effect on spinal kinematics. Unridden exercise is commonly used during early rehabilitation [5] and may also provide a useful alternative/cross-training stimulus for ridden horses. The present findings support previous work demonstrating that walking over poles mobilises the thoracolumbar spine [12,13,21,22], which may contribute to increased activation of the rectus abdominis and longissimus dorsi muscles [10]. This may be particularly relevant in rehabilitation, where horses are often limited to walking exercise and baseline muscle activity is low [32,33].

It is important to highlight the observed increase in maximal thoracic extension when walking over poles, particularly at 100% and 105% spacing. This should be considered in horses where increased spinal extension may be undesirable, such as those with suboptimal posture or pathology involving the dorsal spinous processes [34]. Conversely, shorter pole spacing (85%) reduced thoracic extension but also reduced fore- and hindlimb protraction–retraction ROM, indicating a trade-off that should be considered when selecting pole configurations. However, these findings have been observed in horses without known spinal pathology, so future work needs to assess the effects on horses which have back pain/spinal pathology to clarify direct application to these populations.

Similar increases in limb flexion ROM have been reported during water treadmill exercise, although this modality can also alter protraction–retraction patterns due to drag forces [28,35,36]. Walking in water has been shown to increase both hindlimb protraction and retraction [35]. In contrast, polework increased limb ROM without substantially altering these parameters, which may be advantageous in rehabilitation contexts where increased retraction is undesirable, while water treadmill exercise may offer greater benefits when enhanced hindlimb protraction is the primary goal. In both cases it appears that increasing limb flexion may also increase extension of the thoracic spine [22,37], so it is important that all kinematic effects during exercise selection and progression to greater pole heights and/or water depths are considered and evaluated based on the needs of the individual horse and its training and/or rehabilitation goals.

Overall, the present findings demonstrate that ground poles can be used to modify limb and spinal kinematics during walking. These biomechanical adaptations may be relevant to training and rehabilitation programmes, particularly where specific movement patterns are desired; however, the extent to which such acute kinematic changes translate into improvements in clinical outcomes, performance, visuomotor control, proprioception, or coordination remains to be established [5,6]. An important consideration in prescribing polework is the intensity and frequency required to achieve a training effect. Progressive loading can be achieved by manipulating pole number, height, repetitions, and session frequency, and it should be tailored to the individual horse and adjusted over time [38]. This study was conducted for walking in a straight line; however, polework is also commonly performed ridden and/or at faster gaits and on circular paths. Understanding how locomotor strategies adapt under these conditions is necessary to further inform exercise prescription and support evidence-based progression. In all cases, correct execution is essential, and the prescribed set up should be evaluated through visual observation to ensure that it achieves the intended biomechanical effect in the individual horse.

### 4.4. Limitations

The pole spacing adaptations used in this study were based on previous research by Clayton [18], which included a small sample of highly trained warmblood dressage horses and may not represent the wider horse population. Most horses adapted to the different pole conditions; however, 105% spacing was the most challenging for this population, with some horses taking additional steps between poles. Further increases in spacing may not have been appropriate for all individuals. Allowing a familiarisation trial at 105% spacing may have improved performance but could also have introduced fatigue effects.

All horses were walked in-hand by a single handler positioned on the left side, which may limit generalisability, as handler influence (e.g., speed, positioning, and consistency of guidance) can affect locomotor patterns. Whilst the methods included the application of Bonferroni corrections, the number of statistical comparisons performed increases the risk of Type I error. Additionally, the relatively small sample size may limit the generalisability of the findings and should be considered when interpreting the magnitude and significance of observed effects.

Kinematic data were collected using skin-mounted markers, which are subject to soft tissue artefacts and potential displacement relative to the underlying skeletal structures [39]. While this is a recognised limitation of marker-based motion analysis, it should be considered when interpreting small differences in joint and spinal kinematics. Despite these limitations, the study provides novel insights into the effects of pole spacing on equine locomotor and spinal kinematics.

## 5. Conclusions

Ground pole exercise at walk influences limb and thoracolumbar kinematics in horses, with effects varying according to pole spacing. Increasing pole spacing did not alter maximum limb protraction or retraction but was associated with small increases in protraction–retraction range of motion and greater thoracic extension. In contrast, a shorter pole spacing reduced limb range of motion and maximal hindlimb protraction, suggesting a potential reduction in hindlimb engagement.

Tarsal range of motion increased across all pole conditions, irrespective of spacing, indicating that polework consistently promotes greater limb excursion. Changes in spinal kinematics were primarily observed in the thoracic region, with increased extension at longer pole distances, while effects on spinal flexion and pelvic motion were limited.

These findings indicate that, in practice, pole spacing should be selected according to the desired biomechanical outcome. If increasing limb range of motion is the primary goal, a range of pole spacings may be appropriate. However, where increased thoracic extension is undesirable, shorter pole distances (e.g., 85% step length) may be preferable. Careful selection of pole spacing may therefore support targeted training and rehabilitation strategies, and these should be monitored to ensure that they meet the needs of the individual horse.

## Figures and Tables

**Table 1:** Mean and standard deviation for limb, spine and pelvic kinematics during walking in-hand over no poles and five ground poles (10 cm high) set at 85%, 100% and 105% of step length. − indicates negative value. ^a^ = significantly different to no poles, ^b^ = significantly different to 85% pole distance.

Variable	No Poles	85% Pole Distance	100% Pole Distance	105% Pole Distance
FL Protraction (°)	25.2 ± 1.6	23.2 ± 1.9	24.8 ± 1.1	25.8 ± 2.5
FL Retraction (°)	16.3 ± 1.9	15.8 ± 2.0	15.9 ± 2.1	16.0 ± 1.7
FL ROM (°)	40.7 ± 2.1	37.7 ± 2.8	40.3 ± 2.5	41.1 ± 1.3 ^b^
HL Protraction (°)	32.6 ± 0.7	32.2 ± 1.0	33.9 ± 1.1 ^b^	33.9 ± 1.0 ^b^
HL Retraction (°)	10.8 ± 1.4	9.0 ± 1.1	9.1 ± 1.4	9.5 ± 1.6
HL ROM (°)	43.4 ± 1.6	41.2 ± 1.7	43.0 ± 1.5 ^b^	43.4 ± 1.8
Tarsal Min (°)	126.0 ± 4.3	100.8 ± 5.3 ^a^	98.6 ± 8.0 ^a^	99.8 ± 4.9 ^a^
Tarsal Max (°)	164.0 ± 3.8	162.9 ± 2.9	162.7 ± 3.1	162.6 ± 2.3
Tarsal ROM (°)	38.0 ± 3.6	62.1 ± 4.3	64.2 ± 6.4	62.8 ± 3.7
T10 AMPMin (°)	−20.3 ± 1.7	−21.5 ± 1.5	−21.4 ± 1.7 ^a,b^	−20.3 ± 2.1 ^a^
T10 AMPMax (°)	−12.4 ± 2.3	−11.9 ± 2.2	−12.1 ± 2.2	−12.4 ± 27
T10 ROM (°)	7.9 ± 1.2	9.6 ± 1.3 ^a,b^	9.4 ± 1.2 ^a^	7.9 ± 1.1 ^a^
T13 AMPMin (°)	−12.0 ± 3.3	−13.0 ± 3.3 ^a^	−13.1 ± 3.3 ^a^	−12.0 ± 3.3 ^a^
T13 AMPMax (°)	−3.3 ± 3.8	−2.9 ± 3.6	−3.1 ± 3.8	−2.6 ± 3.7
T13 ROM (°)	8.7 ± 1.3	10.1 ± 1.7	10.1 ± 1.7 ^a^	9.4 ± 1.4 ^a^
T18 AMPMin (°)	3.5 ± 1.8	2.8 ± 2.5	2.6 ± 2.2 ^a^	3.1 ± 2.2 ^a^
T18 AMPMax (°)	11.1 ± 2.5	11.1 ± 2.4	11.3 ± 2.6	11.9 ± 2.7
T18 ROM (°)	7.6 ± 1.2	8.4 ± 1.5	8.7 ± 1.5	8.8 ± 1.5
L3 AMPMin (°)	2.6 ± 1.2	2.0 ± 2.2	1.8 ± 2.0	2.1 ± 1.9
L3 AMPMax (°)	9.6 ± 1.4	9.6 ± 1.5	9.6 ± 1.5 ^a^	10.3 ± 1.8 ^a^
L3 ROM (°)	7.0 ± 1.0	7.6 ± 1.3	7.8 ± 1.3	8.3 ± 1.3
L5 AMPMin (°)	2.8 ± 1.9	2.3 ± 2.2	2.5 ± 1.9	2.8 ± 2.1
L5 AMPMax (°)	9.2 ± 1.5	9.2 ± 1.5 ^a^	9.3 ± 1.5 ^a^	9.9 ± 1.7 ^a^
L5 ROM (°)	6.4 ± 1.0	6.9 ± 1.3	6.8 ± 1.0	7.1 ± 1.6
Pelvic Pitch ROM (°)	8.0 ± 1.2	8.5 ± 1.5	8.1 ± 1.4	7.8 ± 1.4
Speed (m/s)	1.6 ± 0.1	1.4 ± 0.1	1.5 ± 0.1	1.5 ± 0.1

## Data Availability

The datasets presented in this article are not readily available because this was not requested as part of the ethics review process and so they cannot be shared publicly. Requests to access the datasets should be directed to Vicki Walker.

## Supplementary Materials

The following supporting information can be downloaded at https://www.mdpi.com/article/10.3390/ani16131938/s1. Table S1: Linear mixed model output for forelimb (FL) and hindlimb (HL) maximum protraction, retraction and protraction-retraction range of motion (ROM), tarsal min (maximal swing flexion), max (maximal extension) and range of motion (ROM). Table S2: Linear mixed model output for spinal AMP Min (Maximum extension angle), AMP Max (Maximum flexion angle), range of motion (ROM) and pelvic pitch ROM.

## Abbreviations

The following abbreviations are used in this manuscript:

ROM	Range of Motion
NP	No Poles
GP	Ground Poles

## References

[1] Wilson J.M., McKenzie E.C., Duesterdieck-Zellmer K.F. (2018). International survey regarding the use of rehabilitation modalities in horses. Front. Vet. Sci..

[2] Davidson E.J. (2016). Controlled Exercise in Equine rehabilitation. Vet. Clin. N. Am. Equine Pract..

[3] Haussler K.K. (2018). Equine manual therapies in sport horse practice. Vet. Clin. N. Am. Equine Pract..

[4] King M.R. (2022). Rehabilitation: Proprioception, incoordination, and paresis. Vet. Clin. N. Am. Equine Pract..

[5] Haussler K.K., King M.R., Peck K., Adair H.S. (2021). The development of safe and effective rehabilitation protocols for horses. Equine Vet. Educ..

[6] Paulekas R., Haussler K.K. (2009). Principles and practice of therapeutic exercise for horses. J. Equine Vet. Sci..

[7] Walker V.A., Tranquille C.A., MacKechnie-Guire R., Spear J., Newton R., Murray R.C. (2022). Effect of ground and raised poles on kinematics of the walk. J. Equine Vet. Sci..

[8] Brown S., Stubbs N.C., Kaiser L.J., Lavagnino M., Clayton H.M. (2015). Swing phase kinematics of horses trotting over poles. Equine Vet. J..

[9] Clayton H.M., Stubbs N.C., Lavagnino M. (2015). Stance phase kinematics and kinetics of horses trotting over poles. Equine Vet. J..

[10] Shaw K., Ursini T., Levine D., Richards J., Adair S. (2021). The effect of ground poles and elastic resistance bands on longissimus dorsi and rectus abdominus muscle activity during equine walk and trot. J. Equine Vet. Sci..

[11] Ursini T., Shaw K., Levine D., Richards J., Adair H.S. (2022). Electromyography of the multifidus muscle in horses trotting during therapeutic exercises. Front. Vet. Sci..

[12] MacKechnie-Guire R. (2022). Do ground and raised poles affect differential rotational movement of the equine thoracolumbosacral spine during straight line locomotion when walking in-hand?. Proceedings of the BEVA Annual Congress, Liverpool, UK, 2022.

[13] MacKechnie-Guire R. (2022). Do ground or raised poles affect differential rotational movement of the equine thoracolumbosacral spine during straight line locomotion when ridden in rising trot?. Proceedings of the BEVA Annual Congress, Liverpool, UK, 2022.

[14] Pfau T., Witte T.H., Wilson A.M. (2005). A method for deriving displacement data during cyclical movement using an inertial sensor. J. Exp. Biol..

[15] Martin P., Cheze L., Pourcelot P., Desquilbet L., Duray L., Chateau H. (2016). Effect of the rider position during rising trot on the horse’s biomechanics (back and trunk kinematics and pressure under the saddle). J. Biomech..

[16] Brassington R., Hardy R., Bye T. (2025). Long-term effects of treatment and management approaches for impinging dorsal spinous processes in ridden horses. Equine Vet. Educ..

[17] British Horse Society (2017). BHS Complete Horsemanship Volume Three.

[18] Clayton H.M. (1995). Comparison of the stride kinematics of the collected, medium, and extended walks in horses. Am. J. Vet. Res..

[19] Van Weeren P.R., McGowan C., Haussler K.K. (2010). Science overview: Development of a structural and functional understanding of the equine back. Equine Vet. J..

[20] Faber M., Schamhardt H., van Weeren R., Johnston C., Roepstorff L., Barneveld A.B. (2000). Basic three-dimensional kinematicsof the vertebral column of horses walking on a treadmill. Am. J. Vet. Res..

[21] Walker V., Zhu R., MacKechnie-Guire R., Deckers I., te Moller N., Tabor G., Winfield J., Murray R., Maddock C. (2025). How does trotting over ground and raised poles alter equine spinal kinematics?. Equine Vet. J..

[22] Walker V., Slosman C., Pickett M., MacKechnie-Guire R., Deckers I., Te Moller N., Tabor G., Winfield J., Murray R., Maddock C. How does walking over ground and raised poles alter equine spinal kinematics?. Proceedings of the 6th ECVSMR Scientific Meeting.

[23] Smit I.H., Bragança F.M.S., Swagemakers J.J.M., Spoormakers T.J.P. Back motion is largely passive in sound horses at walk. Proceedings of the ICEL9, International Canine and Equine Locomotion Conference.

[24] Hobbs S.J., St George L., Reed J., Stockley R., Thetford C., Sinclair J., Williams J., Nankervis K., Clayton H.M. (2020). A scoping review of determinants of performance in dressage. PeerJ.

[25] Gmel A.I., Haraldsdóttir E.H., Bragança F.M.S., Cruz A.M., Neuditschko M., Weishaupt M.A. (2022). Determining objective parameters to assess gait quality in Franches-Montagnes horses for ground coverage and over-tracking—Part 1: At walk. J. Equine Vet. Sci..

[26] Klimke R. (1985). Basic Training of the Young Horse.

[27] Nankervis K.J., Tranquille C.A., Chojnacka K., Tacey J.B., Deckers I., Newton J.R., Murray R.C. (2023). Effect of speed and water depth on limb and back kinematics in Thoroughbred horses walking on a water treadmill. Vet. J..

[28] Tranquille C., Tacey J., Walker V., Mackechnie-Guire R., Ellis J., Nankervis K., Newton R., Murray R. (2022). Effect of water depth on limb and back kinematics in horses walking on a water treadmill. J. Equine Vet. Sci..

[29] Clayton H.M., White A.D., Kaiser L.J., Nauwelaerts S., Lavagnino M., Stubbs N.C. (2010). Hindlimb response to tactile stimulation of the pastern and coronet. Equine Vet. J..

[30] Clayton H.M., White A.D., Kaiser L.J., Nauwelaerts S., Lavagnino M., Stubbs N.C. (2008). Short-term habituation of equine limb kinematics to tactile stimulation of the coronet. Vet. Comp. Orthop. Traumatol..

[31] Hardeman A.M., Byström A., Roepstorff L., Swagemakers J.H., van Weeren P.R., Serra Bragança F.M. (2020). Range of motion and between-measurement variation of spinal kinematics in sound horses at trot on the straight line and on the lunge. PLoS ONE.

[32] Zsoldos R.R., Kotschwar A., Kotschwar A.B., Rodriguez C.P., Peham C., Licka T. (2010). Activity of the equine rectus abdominis and oblique external abdominal muscles measured by surface EMG during walk and trot on the treadmill. Equine Vet. J..

[33] Robert C., Valette J.P., Pourcelot P., Audigie F., Denoix J.M. (2002). Effects of trotting speed on muscle activity and kinematics in saddlehorses. Equine Vet. J..

[34] Shakeshaft A., Tabor G. (2020). The effect of a physiotherapy intervention on thoracolumbar posture in horses. Animals.

[35] Nankervis K.J., Lefrancois K. (2018). A comparison of protraction-retraction of the distal limb during treadmill and water treadmill walking in horses. J. Equine Vet. Sci..

[36] McCrae P., Bradley M., Rolian C., Léguillette R. (2021). Water height modifies forelimb kinematics of horses during water treadmill exercise. Comp. Exerc. Physiol..

[37] Nankervis K.J., Finney P., Launder L. (2016). Water depth modifies back kinematics of horses during water treadmill exercise. Equine Vet. J..

[38] Castejón-Riber C., Riber C., Rubio M.D., Agüera E., Muñoz A. (2017). Objectives, principles, and methods of strength training for horses. J. Equine Vet. Sci..

[39] van Weeren P.R., van den Bogert A.J., Barneveld A. (1992). Correction models for skin displacement in equine kinematics gait analysis. J. Equine Vet. Sci..

