# Supplementary material for: Does the Distance Between Ground Poles Affect Limb, Spinal and Pelvic Kinematics in Horses When Walking In-Hand?
Source: Animals (Basel). 2026 Jun 23;16(13):1938. doi: 10.3390/ani16131938 (PMC13359845; doi:10.3390/ani16131938)
Supplement: Supplementary file 1 [file animals-16-01938-s001.zip › animals-4350102-supplementary.pdf]

Supplementary Table S1: Linear mixed model output for forelimb (FL) and hindlimb (HL) maximum protraction, retraction and protraction-retraction range of motion (ROM), tarsal min (maximal swing flexion), max (maximal extension) and range of motion (ROM). Conditions significantly different from baseline (No poles) are seen in bold. Significance is set at <0.05.

Variable	Parameter	Estimate	Std Error	df	t	p value	95% CI Lower	95% CI Upper
**FL Protraction**	***Intercept***	*22.31*	*11.03*					
	**85%**	-1.78	2.19	36.29	-0.81	0.42	-6.23	2.66
	**100%**	-0.44	1.79	32.31	-0.25	0.81	-4.09	3.20
	**105%**	-0.85	1.76	31.91	-0.48	0.63	-4.44	2.75
**FL Retraction**	***Intercept***	*11.29*	*3.28*					
	**85%**	0.20	0.61	32.61	0.33	0.75	-1.05	1.45
	**100%**	0.04	0.49	30.76	0.07	0.94	-0.96	1.03
	**105%**	0.09	0.48	30.59	0.19	0.85	-0.88	1.07
**FL ROM**	***Intercept***	*37.93*	*4.97*					
	**85%**	**-2.68**	**1.17**	**38.61**	**-2.29**	**0.03**	**-5.04**	**-0.31**
	**100%**	-0.20	1.02	33.96	-0.20	0.85	-2.28	1.88
	**105%**	0.60	1.01	33.46	0.60	0.55	-1.45	2.66
**HL Protraction**	***Intercept***	*29.54*	*3.61*					
	**85%**	-0.62	0.61	33.19	-1.02	0.32	-1.87	0.62
	**100%**	0.89	0.52	30.22	1.74	0.09	-0.16	1.95
	**105%**	0.86	0.49	29.09	1.76	0.09	-0.14	1.87
**HL Retraction**	***Intercept***	*3.53*	*3.68*					
	**85%**	-0.95	0.62	31.67	-1.55	0.13	-2.21	0.30
	**100%**	-1.15	0.52	27.75	-2.24	0.30	-2.21	-0.10
	**105%**	-0.93	0.49	26.37	-1.89	0.07	-1.94	0.08
**HL ROM**	***Intercept***	*30.63*	*3.71*					
	**85%**	-0.68	0.59	29.69	-1.15	0.26	-1.90	0.53
	**100%**	0.53	0.49	27.58	1.09	0.28	-0.47	1.53
	**105%**	0.72	0.46	26.84	1.56	0.13	-0.23	1.67
**Tarsal Min**	***Intercept***	*141.62*	*13.71*					
	**85%**	**-26.95**	**2.36**	**32.68**	**-11.43**	**<0.001**	**-31.74**	**-22.15**
	**100%**	**-28.49**	**1.99**	**28.09**	**-14.30**	**<0.001**	**-32.57**	**-24.41**
	**105%**	**-27.03**	**1.91**	**26.44**	**-14.17**	**<0.001**	**-30.95**	**-23.11**
**Tarsal Max**	***Intercept***	*149.19*	*6.46*					
	**85%**	0.66	1.03	29.29	0.64	0.52	-1.44	2.76
	**100%**	-0.23	0.84	27.51	-0.27	0.79	-1.95	1.49
	**105%**	-0.58	0.79	26.88	-0.73	0.47	-2.21	1.05
**Tarsal ROM**	***Intercept***	*1.86*	*9.06*					
	**85%**	**28.27**	**2.02**	**35.00**	**13.96**	**<0.001**	**24.16**	**32.38**
	**100%**	**28.66**	**1.85**	**35.00**	**15.52**	**<0.001**	**24.91**	**32.41**
	**105%**	**26.77**	**1.81**	**35.00**	**14.81**	**<0.001**	**23.10**	**30.43**

Supplementary Table S2: Linear mixed model output for spinal AMP Min (Maximum extension angle), AMP Max (Maximum flexion angle), range of motion (ROM) and pelvic pitch ROM. Conditions significantly different from baseline (No poles) are seen in bold. Significance is set at <0.05.

Variable	Parameter	Estimate	Std Error	df	t	p value	95% CI Lower	95% CI Upper
**T10 AMP Min**	***Intercept***	*-15.09*	*2.13*					
	**85%**	-0.71	0.39	30.64	-1.85	0.07	-1.50	0.07
	**100%**	**-1.63**	**0.30**	**29.81**	**-5.38**	**<0.001**	**-2.25**	**-1.01**
	**105%**	**-1.55**	**0.30**	**29.73**	**-5.21**	**<0.001**	**-2.15**	**-0.94**
**T10 AMP Max**	***Intercept***	*-18.71*	*2.59*					
	**85%**	0.86	0.46	30.44	1.86	0.07	-0.09	1.81
	**100%**	0.96	0.36	29.71	2.62	0.10	0.21	1.70
	**105%**	0.73	0.36	29.64	2.05	0.05	0.00	1.47
**T10 ROM**	***Intercept***	*-3.69*	*1.72*					
	**85%**	**1.58**	**0.33**	**34.66**	**4.79**	**<0.001**	**0.91**	**2.26**
	**100%**	**2.59**	**0.27**	**31.78**	**9.75**	**<0.001**	**2.05**	**3.13**
	**105%**	**2.29**	**0.26**	**31.51**	**8.75**	**<0.001**	**1.75**	**2.82**
**T13 AMP Min**	***Intercept***	*-2.37*	*1.90*					
	**85%**	**-1.31**	**0.30**	**29.28**	**-4.36**	**<0.001**	**-1.92**	**-0.69**
	**100%**	**-1.70**	**0.23**	**29.14**	**-7.29**	**<0.001**	**-2.18**	**-1.23**
	**105%**	**-1.75**	**0.23**	**29.13**	**-7.62**	**<0.001**	**-2.21**	**-1.28**
**T13 AMP Max**	***Intercept***	*-7.83*	*2.37*					
	**85%**	-0.01	0.39	29.37	-0.03	0.98	-0.80	0.78
	**100%**	0.09	0.30	29.19	0.31	0.76	-0.52	0.71
	**105%**	-0.08	0.30	29.17	-0.26	0.80	-0.68	0.53
**T13 ROM**	***Intercept***	*-4.95*	*2.36*					
	**85%**	**1.22**	**0.45**	**34.37**	**2.71**	**0.01**	**0.31**	**2.14**
	**100%**	**1.76**	**0.36**	**31.60**	**4.86**	**<0.001**	**1.02**	**2.50**
	**105%**	**1.63**	**0.36**	**31.34**	**4.59**	**<0.001**	**0.91**	**2.36**
**T18 AMP Min**	***Intercept***	*12.80*	*2.55*					
	**85%**	-0.90	0.46	30.28	-1.97	0.06	-1.83	0.03
	**100%**	**-1.03**	**0.36**	**29.58**	**-2.86**	**0.01**	**-1.76**	**-0.29**
	**105%**	**-1.16**	**0.35**	**29.51**	**-3.29**	**<0.001**	**-1.88**	**-0.44**
**T18 AMP Max**	***Intercept***	*10.92*	*1.95*					
	**85%**	-0.60	0.33	29.58	-1.79	0.08	-1.28	0.09
	**100%**	-0.66	0.26	29.29	-2.54	0.20	-1.19	-0.13
	**105%**	-0.54	0.26	29.26	-2.12	0.40	-1.06	-0.02
**T18 ROM**	***Intercept***	*-0.76*	*2.88*					
	**85%**	0.15	0.56	35.24	0.27	0.79	-0.99	1.29
	**100%**	0.28	0.45	31.88	0.63	0.54	-0.64	1.21
	**105%**	0.54	0.45	31.55	1.22	0.23	-0.36	1.45
**L3 AMP Min**	***Intercept***	*9.90*	*2.64*					
	**85%**	-0.55	0.48	30.96	-1.14	0.26	-1.53	0.43
	**100%**	-0.64	0.38	29.96	-1.70	0.10	-1.41	0.13
	**105%**	-0.75	0.37	29.87	-2.02	0.05	-1.50	0.01
**L3 AMP Max**	***Intercept***	*9.23*	*1.71*					
	**85%**	-0.55	0.31	30.25	-1.79	0.08	-1.17	0.08
	**100%**	**-0.66**	**0.24**	**29.61**	**-2.74**	**0.01**	**-1.14**	**-0.17**
	**105%**	**-0.63**	**0.23**	**29.55**	**-2.67**	**0.01**	**-1.11**	**-0.15**

**L3 ROM**	***Intercept***	*1.41*	*2.75*					
	**85%**	-0.28	0.55	36.33	-0.52	0.61	-1.39	0.83
	**100%**	-0.17	0.45	32.17	-0.37	0.71	-1.08	0.75
	**105%**	-0.02	0.44	31.75	-0.04	0.96	-0.92	0.88
**L5 AMP Min**	***Intercept***	*8.17*	*2.62*					
	**85%**	-0.78	0.48	30.86	-1.64	0.11	-1.75	0.19
	**100%**	-0.87	0.37	29.91	-2.34	0.30	-1.64	-0.11
	**105%**	-0.70	0.37	29.82	-1.90	0.07	-1.45	0.05
**L5 AMP Max**	***Intercept***	*9.90*	*1.69*					
	**85%**	**-0.73**	**0.30**	**30.27**	**-2.42**	**0.02**	**-1.35**	**-0.11**
	**100%**	**-0.71**	**0.24**	**29.62**	**-2.99**	**0.01**	**-1.19**	**-0.22**
	**105%**	**-0.60**	**0.23**	**29.56**	**-2.57**	**0.02**	**-1.07**	**-0.12**
**L5 ROM**	***Intercept***	*2.07*	*2.82*					
	**85%**	0.00	0.58	37.70	0.00	1.00	-1.18	1.18
	**100%**	0.14	0.49	33.43	0.29	0.77	-0.85	1.13
	**105%**	0.08	0.48	32.98	0.16	0.88	-0.90	1.05
